# Factors associated with vaccine adherence among an underserved population: the adult Travellers in Nouvelle-Aquitaine, France

**DOI:** 10.1093/eurpub/ckad203

**Published:** 2023-11-29

**Authors:** Sahar Haidar, Elodie Richard, Sophie Vaux, Cecile Allaire, Christine Castor, Daniel Levy Bruhl, Aude Mondeilh, Stéphanie Vandentorren

**Affiliations:** Santé Publique France, French National Public Health Agency, Saint-Maurice, France; Bordeaux Population Health Laboratory, Inserm U1219, University of Bordeaux, Bordeaux, France; Santé Publique France, French National Public Health Agency, Saint-Maurice, France; Santé Publique France, French National Public Health Agency, Saint-Maurice, France; Santé Publique France, French National Public Health Agency, Saint-Maurice, France; Santé Publique France, French National Public Health Agency, Saint-Maurice, France; Fédération Nationale des Associations Solidaires d’Action avec les Tsiganes et les Gens du Voyage (FNASAT—Gens du Voyage), Paris, France; Santé Publique France, French National Public Health Agency, Saint-Maurice, France; Bordeaux Population Health Laboratory, Inserm U1219, University of Bordeaux, Bordeaux, France

## Abstract

**Background:**

A measles epidemic affected the Nouvelle-Aquitaine region from November 2017 to May 2018 with clusters among Travellers. This indicates that measles vaccination rates among Travellers remain lower than in the general population. The objective of this study was to estimate the ‘declarative vaccination’ against measles, mumps and rubella (MMR) and to propose a conceptual framework to help identify determinants of MMR vaccination uptake among adult Travellers in Nouvelle-Aquitaine in 2019–20.

**Methods:**

A cross-sectional study using random sampling was performed and included 612 adult Travellers from 1 November 2019 to 31 March 2020. A conceptual framework to model vaccination adherence was tested among this underserved population by using structural equation modelling. This model included five latent variables: health literacy, attitudes toward preventive measures, stigma, accessibility to care and perceived needs and five measured variables: information received on vaccination, perception of barriers, support for administrative documents, social support and housing conditions.

**Results:**

Individuals who did not answer all the questions linked to the variables included in the model were excluded, thus 347 adults were included in the final sample. The declared vaccination rate against MMR was 74.0%, and 72.4% of the participants were favorable to vaccination. Vaccination adherence was significantly correlated with favorable attitudes toward preventive measures such as having a history of MMR vaccination and not having already refused a recommended vaccine and finally satisfactory information received on vaccination.

**Discussion:**

To improve vaccination adherence, health authorities should lean on personal history with vaccination and on transmitting information on vaccination.

## Introduction

The term Travellers refers to those who live and move around in mobile dwellings or those likely to be mobile, for all or part of the year, i.e. nomads and sedentary people who claim to be travellers. Approximately 250 000–300 000 people were recorded in France in 2019 (6–8 million in Europe).^1^ Their health status is worse than that of the general population^2^ and they have difficulty accessing health care. A national study that collected census and health data from 10 618 Traveller families in Ireland in 2010 found that they had a mortality rate 3.5 times higher than the general population.^3^ The 1987 national study of Travellers’ health status in Ireland reported a lower life expectancy for Irish Travellers: women 11.9 years and men 9.9 years lower than the non-Traveller population.^4^ The use of healthcare and in particular vaccination coverage remained lower than in the general population.^5^ Insufficient vaccination coverage can explain the occurrence of epidemics, such as the measles outbreak in Nouvelle-Aquitaine in 2017, where many clusters occurred among Travellers.^5^ People born since 1980 should have received two doses of the trivalent vaccine available in France, regardless of the history of the three diseases.^6^ No accurate data were available in France, not only concerning the estimation of measles vaccination coverage among adult Travellers but also concerning the factors that could influence their motivation and adherence to vaccination.^5^ However, Travellers are subject to a combination of economic, social and cultural determinants that may influence their motivation to seek vaccination.

Multiple determinants are involved in vaccine intention and adherence. Behavioral theories include the notions of severity and vulnerability, the confidence in preventive measures; the perception of the usefulness of vaccination, social norms and other contextual determinants. Contextual situations are an issue for underserved populations. Thus, the theoretical conceptual model of Andersen and Newman in 2000^7^ (Supplementary appendix S1) takes into account predisposing factors, limiting factors and healthcare needs that influence health behaviors, i.e. hesitancy, adherence and intention to vaccinate, and ultimately the use of vaccination. According to the literature, predisposing factors such as age, gender and education level are major factors in vaccine adherence. Attitudes toward preventive measures are a key construct in the decision-making process^8^ and include having a personal history of vaccination or perceived susceptibility to disease^9^ health literacy, that is associated with knowledge, motivation, and skills in applying health information to make daily decisions regarding care.^8^^,^^10^^,^^11^ Stigma also affects vaccine adherence.^10^^,^^12^ The factors limiting healthcare utilization include geographic accessibility (the ability to access health services), financial or economic accessibility,^13^ and digital accessibility.^14^ Information received is also a factor in vaccine adherence and can also be influenced by attitudes toward preventive measures.^7^ Similarly, the lack of support for administrative procedures is also a limiting factor that affects the motivation to use vaccination. Finally, perceived health needs also influence the motivation and vaccine adherence.^7^ The objective of this study was to estimate the ‘declarative vaccination’ against measles, mumps and rubella (MMR) and to propose a conceptual framework to help identify determinants of MMR vaccination uptake among adult Travellers in Nouvelle-Aquitaine in 2019–20.

## Methods

### Population and study design

The study on the health status and healthcare use of Travellers in Nouvelle-Aquitaine is a cross-sectional study, conducted from 1 November 2019 to 31 March 2020, and then from 15 October 2021 to 31 March 2022 (after an interruption due to the COVID-19 health crisis and France’s lockdown). We chose to focus only on the first phase of this study because the impact of the COVID-19 crisis could influence the results especially the vaccine adherence and vaccine hesitancy.

The first phase included 612 adults and 211 children and the second phase included 418 adults and 126 children. The inclusion criteria were being an adult Travellers (18 years and older), living or had lived in mobile residences, residing in the four selected departments (Gironde, Charente-Maritime, Charente and Creuse) and being known by the local associations of the network of the National Federation of Solidarity Associations for Action with Gypsies and Travelers (FNASAT). The choice of these departments was based on several criteria: the density and diversity of Traveller families, the presence of associations and the consideration of territorial contrasts. The exclusion criteria were the lack of proficiency in the French language and having a place of residence unknown to the associations.

A complex three-stage random sample design was carried out, with the first stage being the living areas (drawn at random by a simple random sample) from among all the living areas in our sampling frame. Living areas were defined by the type of housing according to the Ethos grid (European Typology on Homelessness and housing exclusion) grouping precarious and illegal housing, precarious housing, inadequate housing and adequate housing (Supplementary appendix S2). For the second stage, households were drawn at random from the total households present in the living areas. Finally, for the third stage, one adult was randomly drawn from all adults present in the household.

### Data collection

A pseudonymized standardized questionnaire was administered face to face by a trained social worker. The questionnaire allowed the collection of variables related to demographic and socio-economic characteristics, working and housing conditions, mobility, accessibility and use of healthcare.

### Estimated declarative MMR vaccination

Full MMR vaccination coverage corresponds to two doses of the vaccine to ensure full protection. Vaccination status was determined by self-reported information provided by the individuals themselves through the following question: ‘Are you vaccinated against measles, mumps, rubella (MMR vaccination) and if yes, how many doses?’.

### Vaccination behaviors

To identify vaccination behavior, the variables hesitation and vaccine adherence were considered.

The vaccine hesitancy variable was collected by asking ‘Have you ever decided to delay a vaccine recommended by your doctor for yourself because you were hesitant to vaccinate?’.

The criterion for vaccine adherence was to be favorable to vaccination. This qualitative binary variable corresponds to the question ‘Do you generally favor (or agree with) vaccination?’.

### Conceptual framework

**Figure 1:**
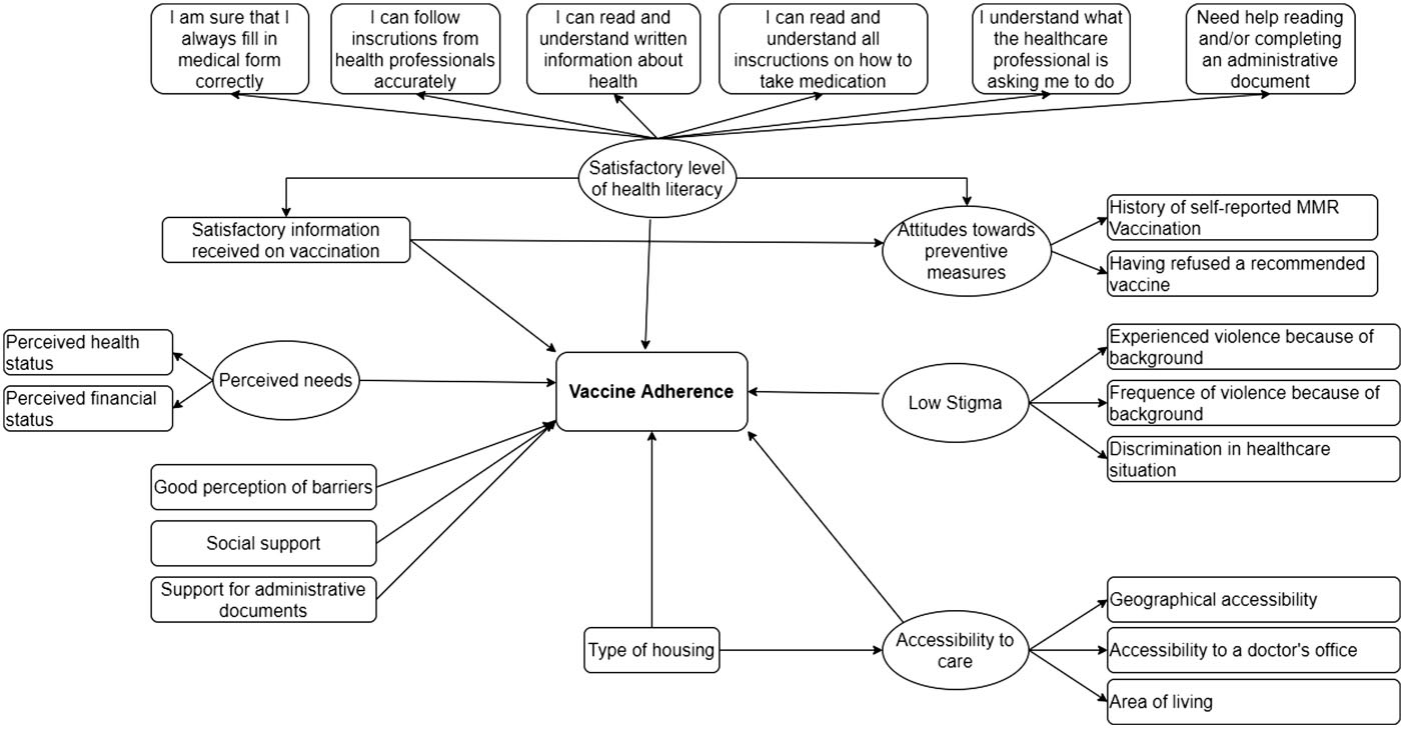
Conceptual model of the determinants of vaccine adherence. Study on Travellers’ use of healthcare and state of health in Nouvelle-Aquitaine in 2019–20

The theoretical conceptual model of Andersen and Newman in 2000^7^ entitled The Behavioral model for vulnerable populations was mobilized. Age, gender and education level were included in the model as confounding factors (figure 1). Our model includes facilitating variables such as health literacy, administrative support, accessibility to care, perceived needs and information received on vaccination, and limiting variables such as attitudes toward preventive measures, stigmatization, and type of housing.

### Construction of the conceptual model

We identified 5 latent variables including 16 indicators (observed variables from the questionnaire) and 5 observed variables not indicative of latent variables (table 1).

**Table 1:** Latent variable construction

Latent variables	No	Indicators	
Attitudes towards preventive measures	1	Self-reported MMR vaccination	No/Yes
	2	Refusing a recommended vaccine	Yes/No
Perceived needs	3	Perceived health status	Poor/Average/Good
	4	Perceived financial status	Debt/Fair/Comfortable
Stigma	5	Experiencing violence because of one's origins	Often/Sometimes/Never
	6	Discrimination in care situations	Yes/No
	7	Frequency of violence due to origins	More than 4 times, 2–3 times, 1 time, 0 times
Access to primary care	8	Geographical accessibility	No/Yes
	9	Accessibility to a doctor	No/Yes
	10	Living area	Rural/Suburban/Urban
Health Literacy	11	I make sure I always fill out the medical forms correctly	Strongly disagree/Somewhat disagree/Somewhat agree/Strongly agree
	12	I can follow instructions from health care professionals accurately	Strongly disagree/Somewhat disagree/Somewhat agree/Strongly agree
	13	I can read and understand written information about health	Strongly disagree/Somewhat disagree/Somewhat agree/Strongly agree
	14	14. I can read and understand all instructions on how to take medication	Strongly disagree/Somewhat disagree/Somewhat agree/Strongly agree
	15	I understand what the health care provider is asking me to do	Strongly disagree/Somewhat disagree/Somewhat agree/Strongly agree
	16	Need help reading and/or completing an administrative document	Yes, I can't read/Yes, I'm not sure I understand/No

### Statistical analysis

Structural equation models (SEM) were used to represent, estimate and test relationships between a set of variables. These variables can be observed variables, i.e. measured in the questionnaire, or unobserved variables, called latent variables, which represent concepts constructed from observed variables. The model included five latent variables (satisfactory level of health literacy, attitudes toward preventive measures, stigmatization, accessibility to care and perceived needs) and five observed variables (satisfactory information received on vaccination, good perception of barriers, social support, support for administrative documents and type of housing).

Each prevalence and mean calculation was weighted and post-stratified on sex. As recommended by the SEM method, we analyzed the weighted covariance matrices of the observed variables of each latent variable. The pairwise correlations had to be >0.30, otherwise the variable was not retained in the model. Next, we checked the unidimensionality of each variable using a ‘Scree-plot’. This allows us to visually assess the number of factors that explain most of the variability in the data and to determine the dimensional structure of a latent variable.^15^ In order to test the correlation between each observed variable and the latent variable to which it belongs, a confirmatory factor analysis was performed.^15^^,^^16^ All estimates were weighted and post-stratified on gender. The goodness of fit of the model was checked by the comparative fit index (CFI > 0.90) and the root mean square error of approximation (RMSEA < 0.08). Analyses were performed on R software version 4.1.0 with the ‘*lavaan-survey*’ packages for SEM estimation and with the WLSMV estimator.

## Results

### Characteristics of the population

The participation rate was 74% (Supplementary appendix S3). Among the 612 participants included in the first phase, 265 people were excluded because they did not answer all the questions linked to the model. Thus, the final sample size was 347 adults. The included individuals for analyses were similar to the excluded study population (Supplementary appendix S6).

The socio-demographic and the housing and mobility characteristics of the participants are presented in table 2. The participants were 42.5 years old on average with a range from 19 to 89 years with 51.7% of women and 48.3% of men. Regarding marital status, those who were in a relationship represented 73.7% of the participants. Regarding education, half (53.9%) of the participants had regular school attendance and had a primary or college education. Of the participants, 8.2% had never attended school. Regarding socio-economic status, more than half (61.6%) of the participants reported being unemployed. Thus, 75.2% were receiving a solidarity income. Concerning the financial situation, 46.4% had a perceived difficult financial situation. The most common type of housing was precarious housing (46.8%) and precarious and illegal housing (31.4%). The caravan remained the most common place of residence for Travellers: 45.5% of the participants declared living in a caravan and 39.9% declared living in mixed housing (caravan and buildings). Indeed, half (58.6%) of the participants travel or change location part of the year and 73.9% of those who travel wish to travel more. Among the reasons cited by those who have travelled less than they would like, we found health problems (52.3%), financial reasons (16.5%), parking problems (12.4%) and children's schooling (10.4%).

**Table 2:** Socio-demographic, mobility and housing conditions characteristics of the study population (*N* = 347), study on Travellers’ use of healthcare and state of health in Nouvelle-Aquitaine in 2019–20

Socio-demographic characteristics	*N*	%^a^	95% CI
Sex	347	100	
Women	234	51.7	
Men	113	48.3	
Age	347	100	
Average (years)	42.5		
18–24 years old	47	9.8	6.7–12.9
25–44 years old	175	50.9	44.1–57.9
45–64 years old	94	31.7	25.2–38.1
≥64 years	31	7.5	4.1–10.9
Family situation	347	100	
In couple	204	73.7	68.4–78.1
Single parent family	60	11.0	7.2–14.8
Single	78	14.4	10.2–18.6
School	347	100	
Never	26	7.6	3.7–11.0
Irregularly	103	38.7	31.6–45.8
Regularly	218	53.9	46.9–60.9
Family situation	347	100	
In couple	235	73.2	68.2–78.2
Single parent family	72	11.1	7.6–14.6
Single	89	14.8	10.8–18.8
Level of education	347	100	
Never	28	8.2	4.4–12.0
Primary education	115	40.6	33.5–47.6
College/specialized institution	169	43.5	36.6–50.4
High school and up	35	7.7	4.8–10.5
Work	280	80.7	
Yes	187	74.1	68.4–79.9
Nature of the work	248	71.4	
Regular	94	36.6	28.7–44.6
Occasional	154	63.4	55.4–71.3
Employment	346	99.7	
You are working	67	27.8	21.9–33.7
You are unemployed	228	61.6	55.6–67.7
You are retired	30	6.48	3.5–9.5
Disability—incapacity	18	3.6	1.9–5.3
Professional status	248	71.5	
Employee	76	20.4	15.5–25.2
Independent	66	39.8	32.8–46.8
Seasonal	92	32.6	26.4–38.8
Not reported	9	4.8	0.7–8.9
Active solidarity income	346	99.7	
Yes	241	75.2	69.8–80.7
Financial status perceived	347	100	
Comfortable	74	18.1	13.8–22.3
Fair	118	35.5	28.6–42.4
Debt	155	46.4	39.2–53.6
Perceived health status	347	100	
Very good or good	192	52.6	45.6–59.7
Average, bad or very bad	155	47.4	40.3–54.4
Mobility and housing characteristics			
Type of housing	347	100	
Adequate/Inadequate	139	21.8	19.4–24.2
Precarious	146	46.8	43.4–50.2
Precarious and illegal	62	31.4	27.9–34.9
Primary housing type	346	99.7	
Appartement/house	14	1.9	0.9–2.9
Construction or similar	92	12.6	10–15.2
Mixed housing (caravan and buildings or similar)	126	39.9	35.0–44.9
Mobile home (caravan)	114	45.5	40.6–50.3
Type of living area	347	100	
Reception or parking area	78	23.3	18.5–28.2
Social or private housing	99	14.6	12.1–17.1
Illegal or precarious parking	56	30.3	26.4–34.2
Family land	84	26.8	21.5–32.1
Rental land	5	1.4	0.3–2.4
Other	25	3.6	1.9–5.3
In the last 5 years, would you say that	341	98.3	
You have not traveled at all	175	37.4	31.3–43.5
You travel all year round	15	3.9	1.7–6.2
You travel part of the year	151	58.6	52.4–64.9
If so, over the past 5 years, would you say that	162	97.6	
You have travelled as much as you wanted to	59	26.1	18.7–33.4
You travelled less than you wanted to	103	73.9	66.6–81.3
If less than you wish, reasons	108	99.0	
Schooling of children	15	10.4	4.3–16.6
Health problems/illness	40	52.3	41.2–63.5
Parking problems	12	12.4	4.9–19.8
Financial reasons	21	16.5	8.8–24.2
Other	14	8.4	3.3–13.4

Note: *N*, number of respondents; 95% CI, 95% confidence interval.

a Weighted and post-stratified proportion on gender.

The characteristics of participants’ access to health care are presented in table 3. Most (97.8%) of the participants had medical coverage; 85% had a complementary health insurance (Solidarity complementary health insurance) and 10.4% had private insurance. Among the participants, 69.4% declared having received help with administrative procedures. Of those who said they had received help, 85.8% had received support for administrative procedures from associations and communal social action centers. Most participants (88.4%) lived near an urban center with health and social services and 72.2% of the homes were served by a transport network.

**Table 3:** Primary care accessibility and vaccination uptake characteristics of the study population (*N* = 347), study on Travellers’ use of healthcare and state of health in Nouvelle-Aquitaine in 2019-2020

Accessibility characteristics	*N*	**%** ^a^	95% CI
Financial accessibility	347	100	
Incomplete	16	4.5	1.9–7.1
Complete	331	95.5	92.9–98.0
Medical coverage	344	99.1	
Yes	336	97.8	95.9–99.7
Supplementary cover	347	100	
None or in progress	16	4.5	1.9–7.1
Solidarity complementary health insurance	286	85.0	80.6–89.4
Private insurance	45	10.4	6.5–14.4
Health mediation: administrative support	347	100.0	
Yes	240	69.4	63.7–75.0
If so, who?	238	99.2	
Association and Communal Center for Social Action	198	85.8	80.9–90.6
Primary Health Insurance Fund	6	1.4	0.3–2.6
Surroundings and family	32	11.7	6–15.6
Proximity to an urban center with health and social services	347	100	
Yes	304	88.4	83.4–93.4
Served by a transportation	347	100	
Yes	216	72.7	68.7–76.8
Having a referring physician	341	98.3	
Yes	307	81.6	75.2–88.1
During the last 12 months, seeing a general practitioner/treating doctor at least once	342	99.0	
Yes	349	85.7	80.5–90.9
Failed to seek treatment when needed	345	99.4	
Yes	58	15.7	11.2–20.2
Characteristics of vaccination uptake			
Perception of a good level of information on vaccination	347	100	
Yes	233	66.6	60.1–73.1
Have a vaccination record	347	100	
Yes	117	26.7	20.7–32.8
None	217	69.1	62.7–75.5
No, but keep an up-to-date personal record	6	1.6	0.1–3.1
Don’t know	7	2.6	0.3–4.8
Support vaccination	347	100	
Somewhat not/Not at all supportive	75	27.6	20.9–34.2
Very/Somewhat favorable	272	72.4	65.8–79.0
Personal refusal of a vaccine recommended by a physician	347	100	
Yes	27	12.3	6.7–17.9
Vaccine hesitation	75	100	
Yes	31	46.8	31.8–61.8
Self-reported MMR vaccination	347	100	
Yes	272	74.0	67.7–80.3
If yes, number of doses	268	98.5	
With 1 dose of vaccine	24	6.9	3.6–10.3
With 2 or more doses of vaccine	90	43.8	36.2–51.4
Don’t know	154	49.3	41.9–56.7
If yes, MMR vaccination in the 2017–18 outbreak?	270	99.3	
Yes with 1 dose	8	2.6	0.6–4.6
Yes with 2 doses	10	4.9	0.3–9.6
None	244	89.0	83.2–94.8
Don’t know	8	3.4	−0.1–6.9
If no, reasons for refusing MMR vaccination	75	100	
Has already had measles	17	19.6	8.9–30.2
Is unfavorable to vaccination	25	43.7	29.3–58.0
Not aware	7	8.9	0.5–17.4
Not proposed by a health professional	11	7.2	2.0–12.3
Other	8	14.1	4.8–23.5
Don’t know	7	6.5	0.6–12.4
Up-to-date vaccination for other diseases	346	99.7	
Yes, I am sure	124	37.9	30.8–44.9
I'm not completely sure or I don't know	124	34.8	28.2–41.3
No, I am not up to date with my vaccinations (at least one vaccine is not up to date)	98	27.3	21.3–33.4
If not up to date, why not?	221	99.5	
Against vaccination	30	20.9	13.0–28.8
Lack of information	37	13.7	8.2–19.2
Not suggested by a health professional	77	29.9	22.1–37.7
Forgot	57	26.2	18.5–33.8
Other	20	9.3	5.8–12.8
Vaccination intention after proposal by doctor today	221	63.7	
No	50	31.9	23.2–40.7
Yes for some vaccines (Hepatitis B, flu)	50	21.7	14.9–28.5
Yes for all vaccines	121	46.3	37.8–54.8

Note: *N*, number of respondents; 95% CI, 95% confidence interval.

a Weighted and post-stratified proportion on gender.

### Vaccination declaration for MMR

For MMR vaccination, nearly three-quarters of participants (74.0%; CI: 67.7–80.3%) declared that they were vaccinated with MMR. Of the 272 adults vaccinated with MMR, 43.8% declared receiving two doses (CI: 36.2–51.4%). The main reasons for those not being vaccinated during the epidemic were being unfavorable to vaccination (43.7%; CI: 29.3–58.0%) and having had measles before (19.6%; CI: 8.9–30.2%).

For the other vaccinations, 27.3% (CI: 21.3–33.4%) of the participants declared their vaccination status not updated because a health professional did not suggest this vaccination (29.9%; CI: 22.1–37.7%), forgetfulness (26.2%; CI: 18.5–33.8%), lack of information (13.7%; CI: 8.2–19.2%) or because they are unfavorable to the vaccination (20.9%; CI: 13.0–28.8%).

### Vaccination behaviors

The characteristics of vaccination use are presented in table 3. Two-thirds of participants reported having a good level of information about vaccination (66.6%). Two-thirds of participants did not have a vaccination record (70.7%).

Regarding vaccine refusal and hesitation, less than half of the participants (12.3%) had refused a vaccine recommended by a doctor and 46.8% had hesitated by delaying a vaccine recommended by a doctor.

Among the participants, 27.6% were unfavorable to vaccination. Of these, 45.7% were against some vaccinations and 44.5% were against all vaccinations.

Regarding vaccine adherence, among the 347 adults in the study sample, 72.4% were somewhat or very favorable to vaccination in general. Vaccination intention for all vaccines in general was 46.3%.

### Measurement model

The weighted correlations between the observed variables of each latent variable ranged from 0.17 to 0.80 (Supplementary appendix S4). “Accessibility to a doctor’s office”, “Discrimination in healthcare situation”, “Perceived health status” and “Perceived financial status” were added back into the model as observed variables not indicative of latent variables. “Perceived needs” were not represented by its chosen indicators and were not maintained. The model fit was acceptable with an RMSEA equal to 0.06 and a CFI equal to 0.73.

### Structural model: factors associated with the use of vaccination

Supplementary appendix S5 presents the final SEM. Vaccination adherence was significantly correlated with favorable attitudes toward preventive measures (β = 0.81, *P* < 0.05). It also improved with information about vaccination (β = 0.12, *P* < 0.05).

## Discussion

### Summary of results

This study shows an MMR-declared vaccination rate estimation of 74.0% which is much lower than the two-dose immunization coverage of 95% expected in order to eliminate the disease,^17^ as it is also the case in the general population (84.0%).^18^ This does not therefore make it possible to stop the transmission of the virus and eliminate the disease.^17^ Vaccination adherence in general, characterized by being favorable to vaccination in general, was 72.4% within the Travellers population in Nouvelle-Aquitaine in 2019–20. Vaccine adherence found among Travellers (72.4%) was of the same order as that of the general population (75.1%) according to the 2016 Health Barometer conducted by telephone between January and August 2016 among 15 216 people aged 15–75 years residing in metropolitan France.^19^ In this study, vaccine adherence was assimilated to being favorable to vaccination in general. Travellers in France had lower vaccination coverage for MMR but not necessarily lower adherence to vaccination in general. This can be due to several factors such as vaccination accessibility which can be harder for nomad Travellers, and the type of vaccines. Travellers are more hesitant to certain vaccines, especially multiple/combined childhood vaccines.^8^

The factors identified as associated with vaccine adherence in our study are attitudes favorable toward preventive measures and satisfactory received information about vaccination. Our results are consistent with the literature. According to the Theory of Planned Behavior model, the intention to get vaccinated depends on a number of predictors, including the attitude toward the vaccine, subjective norms for carrying out vaccination and perception of behavioral control of vaccination.^20^ Attitude is defined as ‘a learned predisposition to respond in a consistently favorable or unfavorable manner’.^21^ It refers to the degree to which a person has a favorable or unfavorable evaluation or appraisal of vaccination. Future vaccination strategies should increase perceived susceptibility to the virus in order to help people form intentions and reduce their vaccine hesitancy.^20^ Although attitudes and norms are notably the stronger predictors of intention, information received by health professionals also have a huge impact on influencing vaccination behavior.^20^ Interventions to improve vaccination should strengthen health information, education and communication (IEC) in order to diminish misperceptions and debunk misinformation. Authorities should work with local partners to coordinate vaccination strategies while taking into consideration the importance of building connections with this community.

### Limitations and strengths of the study

The limit of our study is that the participants included in our study were Travellers known to the local associations of the FNASAT network. Also, factors such as perceived benefits, perceived risks and trust in the health authorities could not be identified because this information were not available in the questionnaire used in this study. However, even though this study can only refer to this population, it presents baseline data on Travellers and allows an initial estimate of vaccination adherence and vaccine uptake among this population in Nouvelle-Aquitaine. In addition, the participation rate was high at 74%. Moreover, the methodology used by the SEMs made it possible to consider all the complex relationships between vaccine adherence and personal and contextual variables. The study identified factors on which vaccination strategies can be based, such as the strengthening of favorable attitudes toward vaccination and the improvement of communication, information and education to vulnerable populations, such as Travellers.

## Conclusion

Targeted actions to promote vaccination among Travellers should encourage actions to promote equality and improve attitudes toward vaccination. Interventions that increase the use of immunization must take into account the vulnerabilities of populations far from the health system by adapting IEC (Information, Education, and Communication) interventions according to the factors identified by this study. This means leaning on strengthening communication with information about vaccination to include these populations.

## Supplementary Material

ckad203_Supplementary_Data

## Data Availability

The data underlying this article will be shared on reasonable request to the corresponding author. Key points The use of healthcare and in particular vaccination coverage remained lower among Travellers than in the general population. Travellers face a combination of economic, social and cultural determinants that influence their motivation to seek vaccination. Vaccination adherence among Travellers is significantly correlated with favorable attitudes toward preventive measures and satisfactory information about vaccination.
