# Supplementary material for: Factors associated with vaccine adherence among an underserved population: the adult Travellers in Nouvelle-Aquitaine, France
Source: Eur J Public Health. 2023 Nov 29;34(1):163–9. doi: 10.1093/eurpub/ckad203 (PMC10843962; doi:10.1093/eurpub/ckad203)
Supplement: ckad203_Supplementary_Data [file ckad203_supplementary_data.docx]

Appendix N˚1: The Behavioral Model for Vulnerable Populations

**
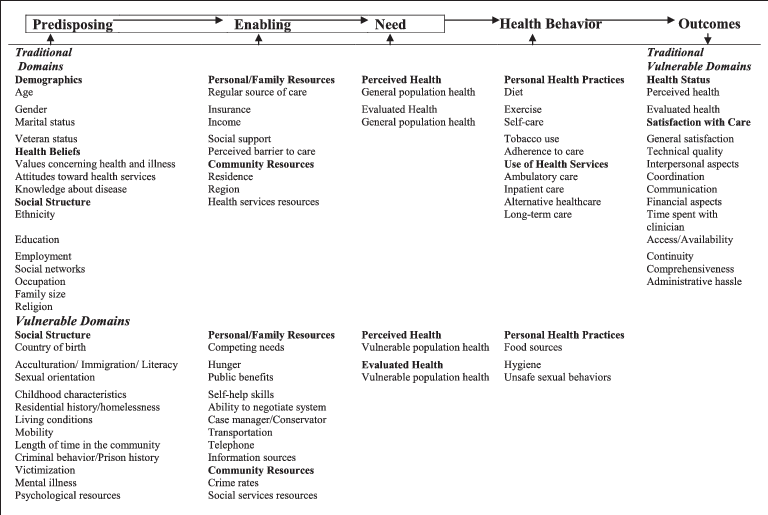
**

Appendix N˚2: Ethos grid (European Typology on Homelessness and housing exclusion).

|  | **NOW (MAJ JANUARY 2020)** | **BEFORE (MAJ OF 05/02/2019)** |
| --- | --- | --- |
| Very precarious housing  (precarious and illegal) | Installation in mobile homes or mixed housing prohibited by national or local planning regulations | Same as |
|  | Installation on a **reception facility** subject to an eviction request | Settlement in a **reception area** subject to a request for eviction. |
|  | **Ordinary housing** in a building or similar prohibited by national or local planning regulations and/or built without permission | **Hard standing** in a building or similar prohibited by national or local planning regulations and/or built without permission |
|  | Installation of a mobile home on a reception facility without a temporary occupation agreement (refused, exceeded or revoked). | Same as |
|  | Installation in a mobile home, mixed housing or **ordinary** housing without the owner's permission | Installation in mobile homes, mixed housing or **permanent housing** without the owner's permission |
|  | Installation of a mobile home on an under-equipped reception facility (halting site, small or large passage area or permanent under-equipped reception area) **with an agreement per household or group** | Installation in a mobile home on an under-equipped reception facility (stopping place, small or large passage area or under-equipped permanent reception area) |
| Precarious housing | Installation of a mobile home on a permanent reception area (not permanent) with an **occupation agreement per household** | Settlement in a mobile home on a permanent reception area (non-permanent) |
|  | Installation of a mobile home or mixed habitat on land belonging to a public (community, State) or private (company, individual) entity with an occupation agreement **for each household** | Installation of a mobile home or a mixed habitat on land belonging to a public (community, State) or private (company, individual) entity with a **temporary or provisional** occupation agreement |
| Inadequate housing | Mobile home, mixed or **ordinary housing** with legal occupancy and secure tenure but equipment standards not met | Mobile homes, mixed housing or **hard standing** with legal occupancy and secure tenure but equipment standards not met |
|  | Mobile home, mixed or **ordinary housing** with legal and secure occupancy but over-occupation | Mobile home, mixed housing or **permanent housing** with legal and secure occupancy but over-occupation |
| Suitable habitat | Mobile home, mixed or **ordinary housing** with legal occupancy, secure, standards respected, no over-occupation | Mobile home, mixed housing or **permanent housing** with legal occupancy, secure, respected standards, without over-occupation |

Appendix N˚3: Flowchart of the number of adults and children included.

Estimated households: **2733**

Eligible households: **1689**

Sampled households

Households not contacted (n=289):

- 124 travels/removals
- 61 imprecise addresses
- 45 unreachable
- 43 end of the survey
- 10 non-travellers
- 6 deaths

Contacted households : **1400**

Investigated living areas: **475**

Included adults: **1030** ^a^

Included children from 7 to 13 years old: **337** ^b^

Households not included (n=370):

- 306 refusals
- 39 absent at the 2^nd^ visit
- 14 already seen in another living area
- 11 language issues

^a^ 612 adults in phase 1 and 418 in phase 2
^b^ 211 children in phase 1 and 126 in phase 2

Appendix N˚4: Measurement model

1. **Validation of latent variables**

The weighted correlations between the indicators of each latent variable ranged from -0.04 to 0.80.

| **Attitudes towards preventive measures** | 1 | 2 |  |  | | |  | |  |
| --- | --- | --- | --- | --- | --- | --- | --- | --- | --- |
| 1. Self-reported MMR vaccination | 1,00 | 0,30 |  |  |  |  | |  |  |
| 2. Refusing a recommended vaccine | 0,30 | 1,00 |  |  |  |  | |  |  |
| **Perceived needs** | 3 | 4 |  |  |  |  | |  |  |
| 3. Perceived health status | 1,00 | 0,19 |  |  |  |  | |  |  |
| 4. Perceived financial status | 0,19 | 1,00 |  |  |  |  | |  |  |
| **Stigma** | 5 | 6 | 7 |  |  |  | |  |  |
| 5. Experiencing violence because of one's origins | 1,00 | 0,20 | 0,80 |  |  |  | |  |  |
| 6. Discrimination in care situations | 0,20 | 1,00 | 0,17 |  |  |  | |  |  |
| 7. Frequency of violence due to origins | 0,80 | 0,17 | 1,00 |  |  |  | |  |  |
| **Access to primary care** | 8 | 9 | 10 |  |  |  | |  |  |
| 8. Geographical accessibility | 1,00 | 0,19 | 0.52 |  |  |  | |  |  |
| 9. Accessibility to a doctor | 0,19 | 1,00 | 0.33 |  |  |  | |  |  |
| 10. Living area | -0,04 | -0,08 | 1.00 |  |  |  | |  |  |
| **Health Literacy** | 11 | 12 | 13 | 14 | 15 | 16 | |  |  |
| 11. I make sure I always fill out the medical forms correctly | 1,00 | 0,44 | 0,39 | 0,46 | 0.31 | 0,36 | |  |  |
| 12. I can follow instructions from health care professionals accurately | 0,44 | 1,00 | 0,38 | 0,44 | 0,60 | 0,24 | |  |  |
| 13. I can read and understand written information about health | 0,39 | 0,38 | 1,00 | 0,77 | 0,27 | 0,37 | |  |  |
| 14. I can read and understand all instructions on how to take medication | 0,46 | 0,44 | 0,77 | 1,00 | 0,37 | 0,35 | |  |  |
| 15. I understand what the health care provider is asking me to do | 0,31 | 0,60 | 0,27 | 0,37 | 1,00 | 0,19 | |  |  |
| 16. Need help reading and/or completing an administrative document | 0,36 | 0,24 | 0,37 | 0,35 | 0,19 | 1,00 | |  |  |

The latent variables "Perceived needs" was rejected because all the correlation coefficients of its indicators were low (below 0.30). The indicators “Accessibility to a doctor’s office”, “Discrimination in healthcare situation”, “Perceived health status” and “Perceived financial status” corresponding respectively to the latent variables healthcare accessibility, stigmatization and perceived needs were rejected from their latent variable, because the correlation coefficients were too low (< 0.30) The unidimensionality of each latent variable was well demonstrated: the scree-plots were all satisfactory (all curves were below 1 from the second point).


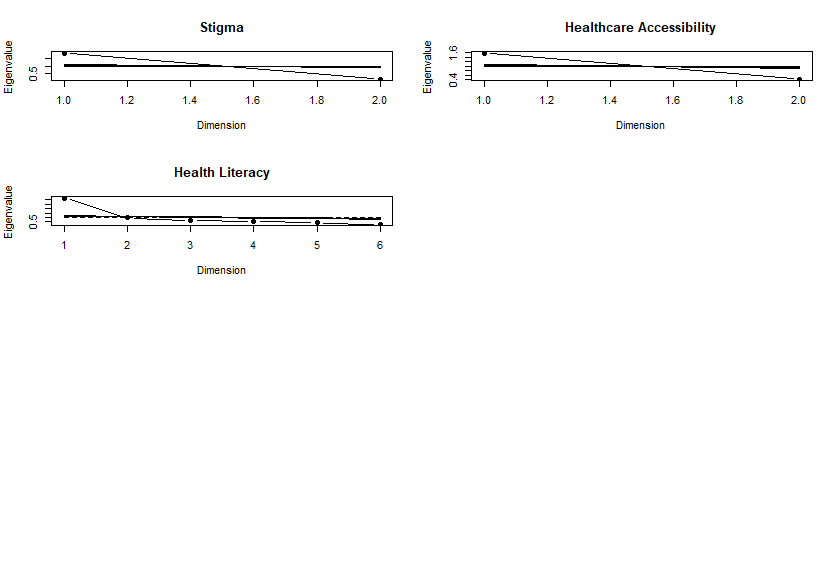


Scree-plot set of latent variables retained in the model. Study on Travellers’ use of healthcare and state of health in Nouvelle-Aquitaine in 2019-2020.

1. **Estimation of the measurement model by confirmatory factor analysis**

Confirmatory factor analysis was used to estimate the relationships between the latent variables and the observed indicator variables. All coefficients were statistically significant (p-value < 0.05). The model fit was acceptable with an RMSEA equal to 0.06 and a CFI equal to 0.73.

Appendix N˚5: **SEM model of the determinants of vaccine adherence among Travellers in Nouvelle-Aquitaine (N=347). Study on Travellers’ use of healthcare and state of health in Nouvelle-Aquitaine in 2019-2020.**


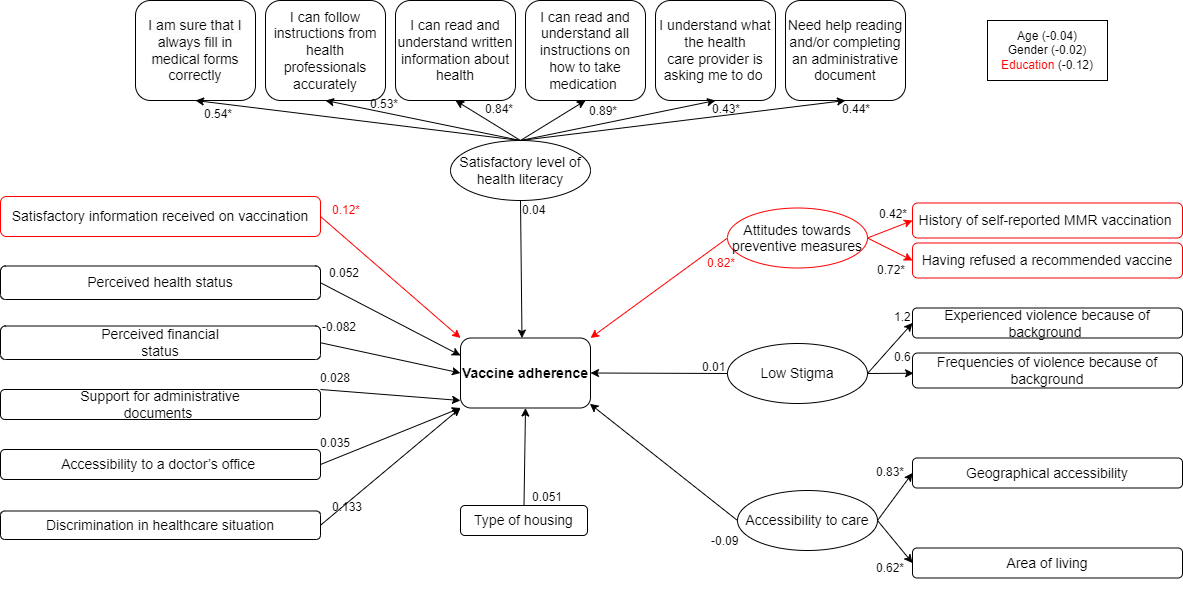


Appendix N˚6: Characteristics of participants with and without missing data. Study on Travellers’ use of healthcare and state of health in Nouvelle-Aquitaine in 2019-2020.

| **Characteristics** | **Population with excluded participants**  **N (%)** | **Population without excluded participants**  **N (%)** |
| --- | --- | --- |
| **Sex** | **612 (100)**  390 (51.7)  222 (48.3) | **347 (100)**  234 (51.7)  113 (48.3) |
| - Woman |  |  |
| - Male |  |  |
| **Age** | **612 (100)** | **347 (100)** |
| *Average (years)* | *43.3* | *42.5* |
| - 18 - 24 years old | 68 (8.9)  297(47.7)  186 (34.7)  61(8.7) | 47 (9.8)  175 (50.9)  94 (31.7)  31 (7.5) |
| - 25 - 44 years old |  |  |
| - 45 - 64 years old |  |  |
| - ≥ 64 years |  |  |
| **Family situation** | **612 (100)**  344 (70.9)  104 (10.7)  155 (16.9)  9 (1.4) | **347 (100)** |
| - In couple |  | 204 (73.7) |
| - Single parent family |  | 60 (11.0) |
| - Alone |  | 78 (14.4) |
| - Other |  | 5 (0.9) |
| **School** | **611 (99.8)**  64 (10.9)  213 (38.7)  334 (50.4 | **347 (100)**  26 (7.6)  103 (38.7)  218 (53.9) |
| - Never |  |  |
| - Irregularly |  |  |
| - Regularly |  |  |
| **Level of education** | **612 (100.0)**  219 (40.1)  278 (41.6)  48 (6.8)  67 (11.5) | **347 (100)**  115 (40.6)  169 (43.5)  35 (7.7)  28 (8.2) |
| - Primary education |  |  |
| - High school/ Specialized institution |  |  |
| - High school and up |  |  |
| - Never |  |  |
| **Work** | **493 (80.5)**  317 (70.3) | **280 (80.7)**  187 (74.1) |
| - Yes |  |  |
| **Nature of the work** | **428 (69,9)**  132 (32.8)  296 (67.2) | **248 (71.4)**  94 (36.6)  154 (63.4) |
| - Regular |  |  |
| - Occasional |  |  |
| **Professional status** | **428 (69.9)**  122 (20.7)  106 (36.9)  175 (35.9)  17 (4.8)  8 (1.7) | **248 (71.5)**  76 (20.4)  66 (39.8)  92 (32.6)  9 (4.8)  **-** |
| - Employee |  |  |
| - Independent |  |  |
| - Seasonal |  |  |
| - Not reported |  |  |
| - Other |  |  |
| **Employment** | **610 (99.7)**  120 (26.6)  384 (59.4)  64 (8.7)  37 (4.9)  5 (0.4) | **346 (99.7)**  67 (27.8)  228 (61.6)  30 (6.48)  18 (3.6)  - |
| - You are working |  |  |
| - You are unemployed |  |  |
| - You are retired |  |  |
| - Disability - Incapacity |  |  |
| - Other |  |  |
| **Active solidarity income** | **608 (99.3)**  415 (72.4) | **346 (99.7)**  241 (75.2) |
| - Yes |  |  |
| **Financial status perceived** | **609 (99.5)**  140 (19.6)  199 (31.8)  269 (48.3) | **347 (100)**  74 (18.1)  118 (35.5)  155 (46.4) |
| - Comfortable |  |  |
| - Just |  |  |
| - You can't get it right/ Debt |  |  |
| **Type of habitat** | **612 (100.0)**  260 (25.2)  245 (46.2)  107 (28.6) | **347 (100)**  139 (21.8)  146 (46.8)  62 (31.4) |
| - Adequate / Inadequate |  |  |
| - Precarious |  |  |
| - Precarious and illegal |  |  |
| **Primary habitat type** | **590 (96.4)**  173 (14.8)  192 (38.5)  199 (44.5)  26 (2.2) | **346 (99.7)**  92 (12.6)  126 (39.9)  114 (45.5)  14 (1.9) |
| - Construction |  |  |
| - Mixed housing (caravan and buildings) |  |  |
| - Mobile home (caravan) |  |  |
| - Other |  |  |
| **Type of living space** | **593 (96.9)**  117 (21.7)  187 (16.8)  95 (27.9)  144 (27.3)  8 (1.3)  42 (5.0) | **347 (100)**  78 (23.3)  99 (14.6)  56 (30.3)  84 (26.8)  5 (1.4)  25 (3.6) |
| - Reception or parking area |  |  |
| - Social or private housing |  |  |
| - Illegal or precarious parking |  |  |
| - Family land |  |  |
| - Rental land |  |  |
| - Other |  |  |
| **In the last 5 years, would you say that** | **600 (98.0)**  327 (41.3)  23 (3.7)  250 (55.0) | **341 (98.3)**  175 (37.4)  15 (3.9)  151 (58.6) |
| - You have not traveled at all |  |  |
| - You travel all year round |  |  |
| - You travel part of the year |  |  |
| **If so, over the past 5 years, would you say that** | **266 (43.5)**  100 (31.3)  166 (68.7) | **162 (97.6)**  59 (26.1)  103 (73.9) |
| - You have travelled as much as you wanted to |  |  |
| - You travelled less than you wanted to |  |  |
| **If less than you wish, reasons** | **161 (96.6)**  25 (12.3)  55 (42.2)  21 (15.1)  33 (17.4)  27 (12.9) | **108 (99.0)**  15 (10.4)  40 (52.3)  12 (12.4)  21 (16.5)  14(8.4) |
| - Schooling of children |  |  |
| - Financial reasons |  |  |
| - Parking problems |  |  |
| - Health problems/illness |  |  |
| - Others |  |  |
| **Medical coverage** | **603 (98.5)**  581 (96.4) | **344 (99.1)**  336 (97.8) |
| - Yes |  |  |
| **Supplementary cover** | **607 (99.2)**  32 (6.2)  488 (82.5)  78 (9.3) | **347 (100)**  16 (4.5)  286 (85.0)  45 (10.4) |
| - None or in progress |  |  |
| - Solidarity complementary health insurance |  |  |
| - Mutual / Private Insurance |  |  |
| **Health mediation: administrative support** | **608 (99.3)**  444 (73.0) | **347 (100.0)**  240 (69.4) |
| Yes |  |  |
| **If so,by who?** | **442 (72.2)**  370 (84.6)  9 (1.2)  8 (1.1) | **238 (99.2)**  198 (85.8)  6 (1.4)  32 (11.7) |
| - Association and Communal Center for Social Action |  |  |
| - Primary Health Insurance Fund |  |  |
| - Surroundings and family |  |  |
| **During the last 12 months, seeing a general practitioner/treating doctor at least once** | **599 (97.9)**  510 (84.1) | **342 (99.0)**  349 (85.7) |
| - Yes |  |  |
| **I'm sure I always fill out the medical forms correctly** | **601 (98.2)**  167 (27.8)  176 (29.1)  147 (22.8)  111 (20.2) | **347 (100)**  80 (22.8)  117 (33.5)  73 (21.3)  77 (22.4) |
| - Strongly disagree |  |  |
| - Somewhat disagree |  |  |
| - Somewhat agree |  |  |
| - Totally agree |  |  |
| **I can follow the instructions of health care professionals accurately** | **601 (98.2)**  45 (6.5)  240 (39.8)  83 (12.9)  233 (40.7) | **347 (100)**  25 (5.2)  140 (43.7)  37 (10.3)  145 (40.7) |
| - Strongly disagree |  |  |
| - Somewhat disagree |  |  |
| - Somewhat agree |  |  |
| - Totally agree |  |  |
| **I can read and understand written health information** | **600 (98.0)**  167 (29.2)  174 (26.7)  119 (21.8)  140 (22.3) | **347 (100)**  88 (27.4)  112 (26.7)  52 (21.8)  95 (24.0) |
| - Strongly disagree |  |  |
| - Somewhat disagree |  |  |
| - Somewhat agree |  |  |
| - Totally agree |  |  |
| **I can read and understand all instructions on how to take medication** | **596 (97.4**  163 (28.9)  161 (23.9)  106 (19.9)  166 (27.1) | **347 (100)**  76 (24.5)  101 (24.1)  59 (21.9)  111 (29.5) |
| - Strongly disagree |  |  |
| - Somewhat disagree |  |  |
| - Somewhat agree |  |  |
| - Totally agree |  |  |
| **I understand what the health professional is asking me to do** | **600 (98.0)**  14 (1.8)  268 (44.1)  45 (8.5)  273 (45.6) | **347 (100)**  9 (1.6)  143 (41.1)  25 (9.5)  170 (47.9) |
| - Strongly disagree |  |  |
| - Somewhat disagree |  |  |
| - Somewhat agree |  |  |
| - Totally agree |  |  |
| **When you have to fill in an administrative document, do you need help to read and/or complete it?** | **398 (98.5)**  101 (14.8)  502 (85**.**2) | **347 (100)**  68 (16.8)  279 (83.2) |
| - No |  |  |
| - Yes |  |  |
| **Perceived health status** | **607 (99.2)**  316 (50.1)  290 (49.8) | **347 (100)**  192 (52.6)  155 (47.4) |
| - Very good or good |  |  |
| - Average, bad or very bad |  |  |

*weighted and post-stratified proportion on gender

N: number of respondents
